# Protective Effects of Spirulina Supplementation on Chondrocytes Under Moderate Acute Dynamic Compression

**DOI:** 10.1177/19476035261430274

**Published:** 2026-03-09

**Authors:** Nadia Golestani, Saba Rafieian, Stephen D. Waldman, Lea Zila, Wendy Pearson

**Affiliations:** 1 Department of Animal Biosciences, University of Guelph, Guelph, ON, Canada; 2 Institute of Biomedical Engineering, University of Toronto, Toronto, ON, Canada; 3 Physical Platform, Holland Bone and Joint Program, Sunnybrook Research Institute, Toronto, ON, Canada; 4 Institute of Biomedical Engineering, Science and Technology (iBEST), Unity Health and Toronto Metropolitan University, Toronto, ON, Canada; 5 Department of Chemical Engineering, Toronto Metropolitan University, Toronto, ON, Canada; 6 Li Ka Shing Knowledge Institute, St. Michael’s Hospital, Toronto, ON, Canada

**Keywords:** Spirulina, compression, chondrocyte, cartilage, nitric oxide

## Abstract

**Objective:**

To determine the influence of Spirulina supplementation on the response of bovine chondrocytes to mechanical compression with respect to extracellular matrix dynamics (proteoglycan and collagen) and inflammation [nitric oxide (NO)].

**Methods:**

Bovine chondrocytes were embedded in agarose constructs and cultured for 14 days in basal media or media supplemented with Spirulina (30 or 90 µg/mL). Constructs were exposed to 10% dynamic compression. DNA content, NO levels, proteoglycan content and release, and collagen synthesis were measured to assess extracellular matrix (ECM) responses.

**Results:**

DNA content did not differ among groups. Spirulina supplementation increased NO levels in constructs and media, with the highest effect observed at 90 µg/mL. Proteoglycan content decreased in Spirulina-treated constructs and declined further after compression, while proteoglycan release increased across all groups. Collagen synthesis and content were elevated by Spirulina, particularly at 90 µg/mL, and further increased following dynamic compression.

**Conclusions:**

Spirulina supplementation, particularly at higher concentrations, enhances ECM turnover and increases NO production in chondrocytes under mechanical loading, indicating potential modulation of matrix dynamics that may be beneficial. However, these results are derived from an *in vitro* model and may not fully represent *in vivo* physiological conditions.

## Introduction

Articular cartilage is a specialized connective tissue covering synovial joint surfaces that enables low-friction articulation and load distribution through an extracellular matrix rich in type II collagen, proteoglycans, and water.^1-5^ Because cartilage is avascular and aneural, its capacity for intrinsic repair is limited, making maintenance of ECM homeostasis critical for long-term joint function.^3,4^

Mechanical loading is a key regulator of cartilage homeostasis, with physiological dynamic loading promoting anabolic ECM synthesis, while excessive or abnormal loading activates catabolic pathways, oxidative stress, and matrix degradation.^1^,^6-9^ NO plays a central role in these mechanotransduction pathways, supporting chondrocyte adaptation under normal loading but contributing to oxidative damage when overproduced.^10-12^ Alterations in synovial fluid composition with aging or osteoarthritis (OA) further modify the mechanical and biochemical environment of cartilage, amplifying susceptibility to load-induced injury.^6,13^ Early OA progression is influenced by mechanical stress, aging, and chronic inflammation, highlighting the need for interventions that preserve ECM integrity.^14^

While mechanobiology is well-characterized, the role of dietary or nutraceutical interventions in modulating cartilage responses to mechanical stress remains underexplored. Spirulina, a cyanobacterium rich in antioxidants such as C-phycocyanin and β-carotene, has been shown to attenuate oxidative stress and inflammatory responses in various tissues.^15,16^ Recent reviews highlight Spirulina’s capacity to modulate redox-sensitive signaling pathways rather than acting as a simple radical scavenger.^17^ Given the dual role of NO as both a physiological mechanotransduction mediator and a marker of pathological stress, Spirulina’s reported effects on oxidative stress and NO regulation suggest a potential role in preserving cartilage health under mechanical loading.^17^

However, studies directly investigating the combined effects of controlled mechanical compression and Spirulina supplementation on chondrocyte inflammatory signaling, ECM turnover, and oxidative stress are lacking. Addressing this gap could inform tissue-engineering strategies and rehabilitation protocols integrating mechanical conditioning with nutritional interventions. The present study addresses this gap by evaluating the effects of Spirulina supplementation on cartilage constructs subjected to dynamic mechanical loading. The objective of this study was to determine whether Spirulina supplementation modulates inflammatory signaling, ECM turnover, and oxidative responses in chondrocytes under controlled mechanical loading, thereby informing future strategies for cartilage preservation and joint health.

## Materials and Methods

All reagents were purchased from Sigma Aldrich (Mississauga, Ontario, Canada) unless otherwise specified. Metacarpophalangeal joints from bovine donors were obtained post-mortem from a federally inspected meat processing facility (Peel Meat Packers, Drayton, Ontario) for the collection of tissue samples used in this study. No live animals were used, and therefore no ethical review or animal care approval was required.

### Spirulina Extract

The Spirulina extract was prepared following a previously established method using a combination of water and ethanol.^18^ The amount of Spirulina powder (Selected Bioproducts Inc., Guelph, Ontario, Canada) added to the tissue culture wells was calculated based on an estimated *in vivo* concentration. This was derived by calculating the estimated concentration of a single dose (15 g) into the total body water compartment of a 500 kg horse (approximately 330 L),^19^ and reproducing this concentration in tissue culture media. This informewd our initial concentration of 30 μg/mL, and we also evaluated the effect of 3 times this concentration (90 μg/mL). While we acknowledge that this approach ignores the influences of bioavailability and pharmacodynamics, they are a reasonable approximation of the lower and higher ends of a biologically relevant range estimated *in vivo* (~45 µg/mL). The chosen concentrations are also consistent with those reported in previous *in vitro* studies evaluating Spirulina’s antioxidant and anti-inflammatory effects, supporting their physiological and experimental relevance.^19^ A blank extract (B) was prepared using the same procedure but without the addition of Spirulina.

### Chondrocyte-Agarose Hydrogel Constructs

Primary bovine chondrocytes were isolated from full-thickness cartilage slices using a sequential enzymatic digestion method, following established protocols. Cartilage from the upper and lower condyles of the metacarpophalangeal joints of bovine donors aged 20-24 months was dissected and subjected to protease treatment for 1 hour, followed by collagenase incubation for 18 hours in Ham’s F12 media.^20^ Chondrocytes were extracted through a 2-step enzymatic process. Initially, the cartilage slices were treated with a 0.5% (w/v) protease solution for 1 hour at 37°C. This was followed by incubation in a 0.15% (w/v) collagenase solution for 18 hours at 37°C in a 5% CO_2_ atmosphere.

Chondrocytes were isolated by filtering the digested solution through a 200-mesh filter to remove undigested particles. Cells were washed thoroughly with Ham’s F12 media and further filtered using a 70 μm cell strainer. Chondrocytes were then centrifuged at 700 × g for 7 min to form a cell pellet. After washing the cells 4 times with Ham’s F12 medium, they were counted using the Trypan blue exclusion method to assess viability.^21^ Viable chondrocytes were subsequently seeded in type VII low melting point agarose, prepared with phosphate-buffered saline (PBS, pH 7.4), resulting in a 2% (w/v) low-gelling agarose (A9045, type VII) with a seeding density of approximately 10 × 10⁶ cells/mL (250,000 cells per construct). The seeds were cast into custom PTFE molds to form cylindrical constructs (3 mm by 3 mm; 2.1 × 105 cells/construct, The constructs were transferred to individual wells of a 24-well plate. Constructs were cultured under static (no-load) conditions for 2 weeks, with media changes every 2 days under normoxic conditions at 37°C and 5% CO_2_. The complete media consisted of Ham’s F12 media containing 20% (v/v) fetal bovine serum (FBS), 20 mM HEPES, 100 μg/mL ascorbic acid, and 2% antibiotics/antimycotics.

### Application of Mechanical Stimulation

After a 2-week pre-culture period, constructs underwent a single bout of sinusoidal compression at 1 Hz with a 10% strain amplitude for 20 min using a custom dynamic compression device. The design and components of the device, including its compatibility with a 6-well plate and its ability to compress up to 16 constructs simultaneously, have been detailed in our previous work.^22,23^

### Harvesting of Chondrocyte-Agarose Hydrogel Constructs

Following mechanical stimulation, ECM biosynthesis was quantified using radioisotope incorporation, as previously described.^23^ To label newly synthesized collagen, [³H]-proline (2.5 μCi/mL) was added immediately after mechanical stimulation to each well, and constructs were incubated at 37°C with 5% CO₂ for 24 hours. After incubation, constructs were washed 3 times with PBS to remove unincorporated isotopes and enzymatically digested in 40 μg/mL papain buffered with 20 mM ammonium acetate, 1 mM EDTA, and 2 mM DTT for 72 hours at 65°C. Digested samples were stored at −20°C until analysis. Media samples were collected before and 24 hours after compression. All biochemical assays, except for DNA content, were performed on media samples as well as constructs. Since proteoglycan and collagen were not detectable in the media, precipitation was performed before analysis. Proteoglycans were precipitated from the culture medium using cold ethanol. Specifically, 100% ethanol was added slowly to the defrosted medium at a 3:1 ratio (ethanol to medium), and samples were incubated overnight at 4°C. The resulting pellet was collected by centrifugation, washed multiple times with cold 70% ethanol, and dried. The pellet was then resuspended in 4 M guanidinium hydrochloride, and proteoglycan content was quantified using a glycosaminoglycan (GAG) assay. Collagen was extracted from the culture medium using ammonium sulfate precipitation. Briefly, 70% ammonium sulfate was added slowly to the defrosted medium (1.5 mL per 2 mL of medium), followed by gentle mixing at 4°C and overnight precipitation. The collagen-containing pellet was collected by centrifugation, washed repeatedly with cold 70% ethanol, and dried. The pellet was then digested with papain, and collagen content was quantified using a hydroxyproline assay. Assays were then conducted as described below.

### Biochemical Assays

DNA content was determined using the Hoechst 33258 DNA assay. NO levels were measured using the Griess Reaction method, which quantifies nitrite (NO₂⁻), a stable oxidation product of NO.^24^ Proteoglycan content was assessed using the 1,9-dimethyl methylene blue (DMB) assay.^25^ The accumulation of newly synthesized collagen was quantified by measuring radioisotope incorporation of the digested sample mixed with scintillation cocktail (ScintiSafe Econo 2 Cocktail, Fisher Scientific) and analyzed using an LS6500 β-liquid scintillation counter (Beckman Coulter). Total collagen content was estimated based on hydroxyproline levels. Papain-digested samples were hydrolyzed in 6 N HCl at 110°C for 18 hours. Hydroxyproline levels in the hydrolysate were determined using the chloramine-T/Ehrlich’s reagent assay.^26^ Collagen content was calculated based on the assumption that hydroxyproline constitutes approximately 10% of collagen’s weight.^27^

### Statistical Analysis

For each experiment, at least 2 separate cell isolations from different animals were utilized to account for donor variability. Results were compiled from 2 independent experiments, with each experiment using cells pooled from 2 to 3 bovine joints. Two trials were conducted. In this design, treatment group (B, T1, T3) and compression (before vs. after) were treated as fixed effects, as these were pre-determined experimental conditions of primary interest. Cell isolation, and experimental batch were considered random effects, representing random samples of potential biological and technical variability. All results were normalized to the average of each group, and combined data were presented as the mean ± standard error of the mean (SEM). A 95% confidence level was assumed for statistical testing. All of the assays were run in duplicate and were normalized to DNA content of each group. Statistical analyses were conducted using GraphPad Prism 10.4.1. Data were analyzed using t-tests, 1-way ANOVA, or 2-way ANOVA with post-hoc Tukey tests. A *P*-value of <0.05 was considered statistically significant.

## Results

Since there was no significant difference between groups B and C (*P* > 0.99), all other groups were compared to group B, which served as the reference control.

### DNA Content

**Figure 1.**
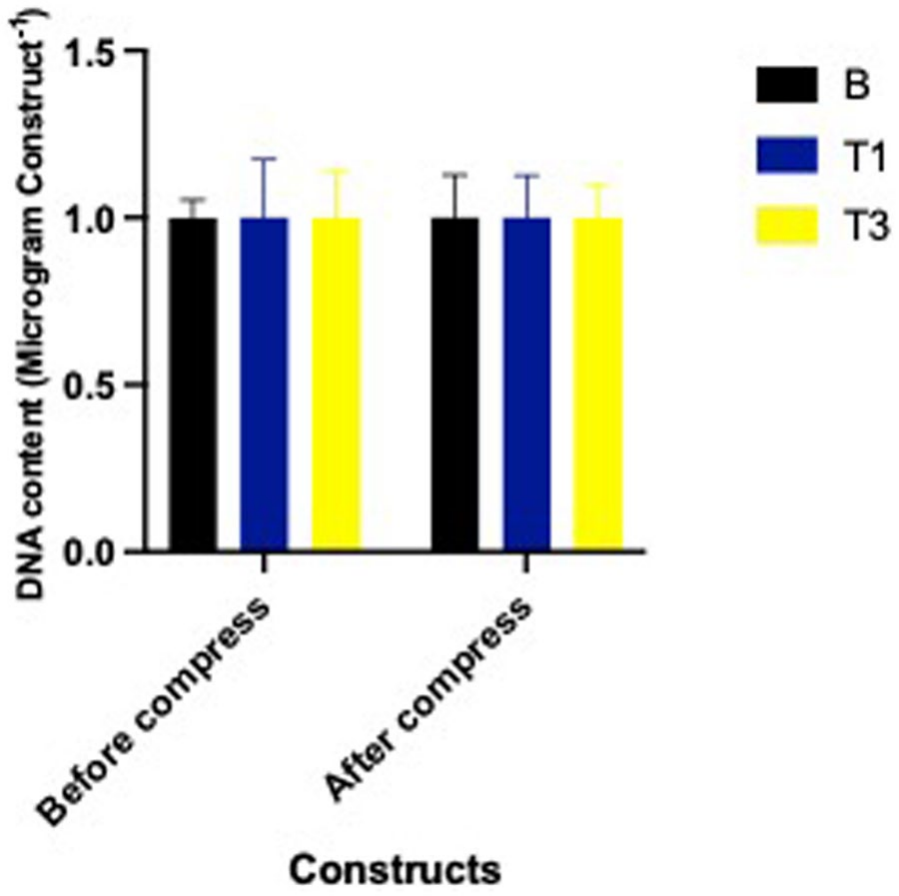
DNA Content (μg/construct) of constructs cultured with Spirulina at 0 (B; carrier only), 30 (T1), and 90 (T3) µg/mL on DNA content before and after a single bout of dynamic compression.

DNA content remained consistent across all treatment groups—B, T1, T3—both before and after mechanical compression (*P* = 0.99). This indicates that neither Spirulina supplementation nor dynamic loading affected cell number or viability within the constructs throughout the experiment **(Fig. 1).**

### NO in Constructs

**Figure 2.**
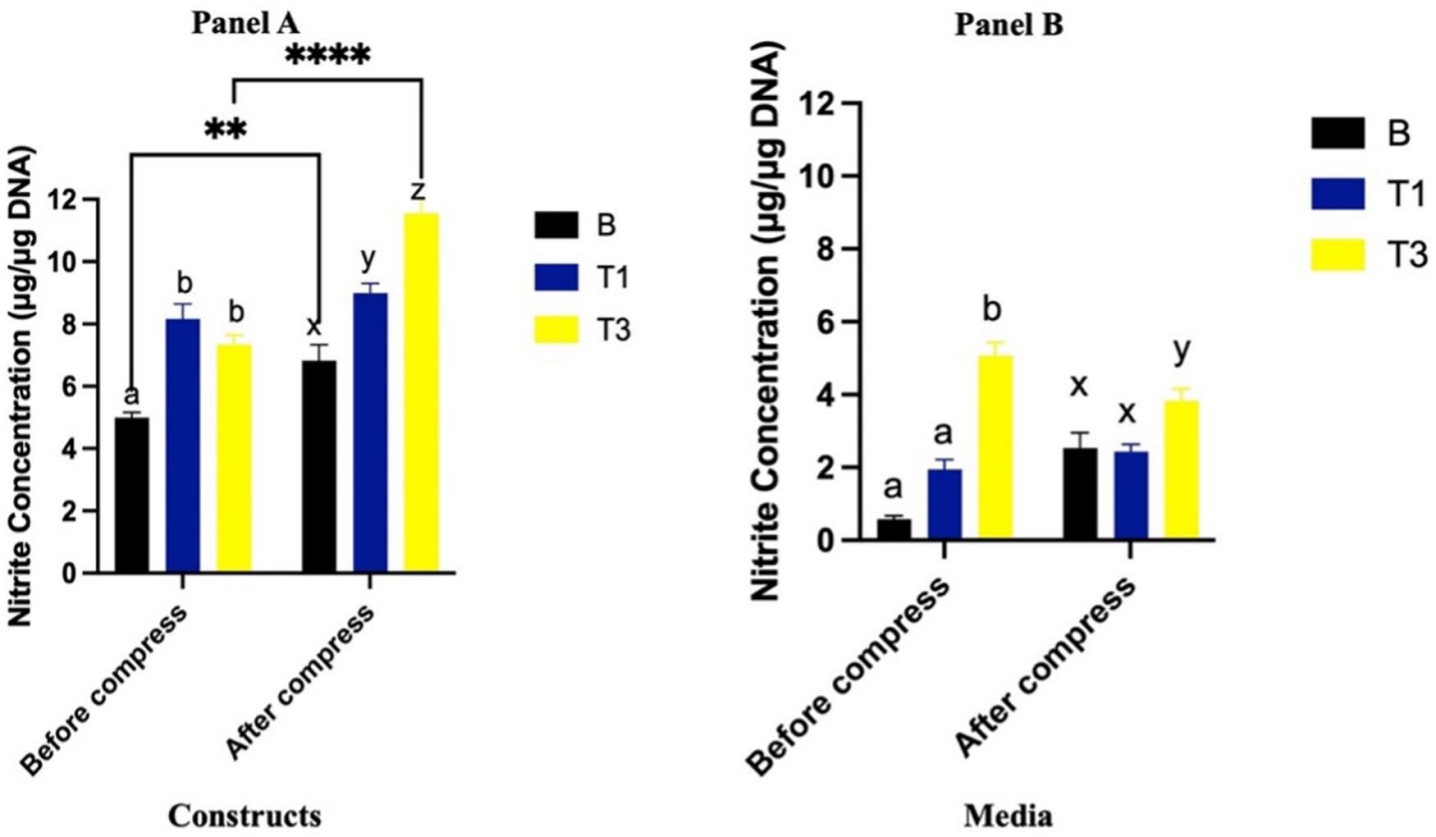
Nitrite content (μg/μg DNA) of constructs (**Panel A**) and media from constructs (**Panel B**) cultured with Spirulina at 0 (B; carrier only), 30 (T1) and 90 (T3) µg/mL before and after a single bout of dynamic compression. Different lowercase letters (abc: pre-compression, xyz: post-compression) denote significant differences between groups before and after compression. *Indicates significant difference between pre- and post-compression within the same group.

Prior to compression, NO in constructs were significantly greater in both T1 and T3 compared to B (*P* < 0.0001), with no significant difference between T1 and T3 (*P* = 0.25) **(Fig 2, panel A).** Following compression, NO increased in the B (*P* = 0.002) and T3 (*P* < 0.0001) groups, while no change was observed in T1 (*P* = 0.15). After compression, NO content remained significantly greater in T1 and T3 compared to B (*P* < 0.002 and *P* < 0.0001, respectively), with T3 exhibiting greater NO than T1 (*P* = 0.0004) **(Fig 2, panel A).**

### NO in Media

Prior to compression, NO in the media was significantly greater in the T3 group compared to both B and T1 (*P* < 0.0001 for both), while no difference was observed between T1 and B (*P* = 0.55). Compression did not result in significant changes in NO in any groups (*P* = 0.55). However, following compression, NO remained greater in T3 compared to B (*P* < 0.0001) and T1 (*P* < 0.0001), with no difference between T1 and B (*P* = 0.22) (**Fig 2, Panel B**).

### Proteoglycan Content in Constructs

**Figure 3.**
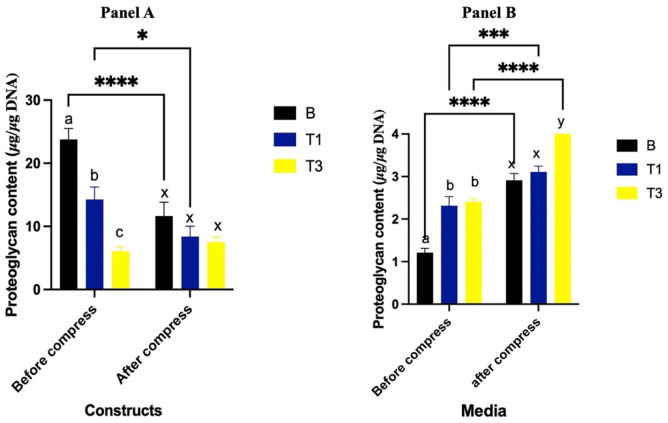
Proteoglycan content (μg/μg DNA) of constructs (**Panel A**) and media from constructs (**Panel B**) cultured with Spirulina at 0 (B; carrier only), 30 (T1) and 90 (T3) µg/mL before and after a single bout of dynamic compression. Different lowercase letters (abc: pre-compression, xyz: post-compression) denote significant differences between groups before and after compression. *Indicates a significant difference between pre- and post-compression within the same group.

Prior to compression, constructs cultured with T1 and T3 had lesser proteoglycan content compared to the B group (*P* = 0.0007 and *P* < 0.0001, respectively) (**Fig 3, Panel A**). T3 constructs had lesser proteoglycan levels than T1 (*P* = 0.0029). Following compression, proteoglycan content significantly decreased in the B group (*P* < 0.0001) and in T1 constructs (*P* = 0.01), while no significant change was observed in the T3 group (*P* = 0.51). After compression, proteoglycan content was not significantly different between groups.

### Proteoglycan Content in Media

Prior to compression, proteoglycan levels in the media were significantly greater in the T1 and T3 groups compared to the B group (*P* < 0.0001 for both) (**Fig 3, Panel B**). Following compression, proteoglycan release in the media increased in the B group (*P* < 0.0001). Similarly, proteoglycan release in the T1 and T3 groups increased compared to pre-compression levels (*P* < 0.0007 and *P* < 0.0001, respectively). Post-compression, proteoglycan release was significantly greater in T3 compared to B (*P* < 0.0001), while no difference was observed between T1 and B (*P* = 0.62). Additionally, T3 had greater proteoglycan content compared to T1 (*P* = 0.0002).

### Collagen Synthesis in Constructs

**Figure 4.**
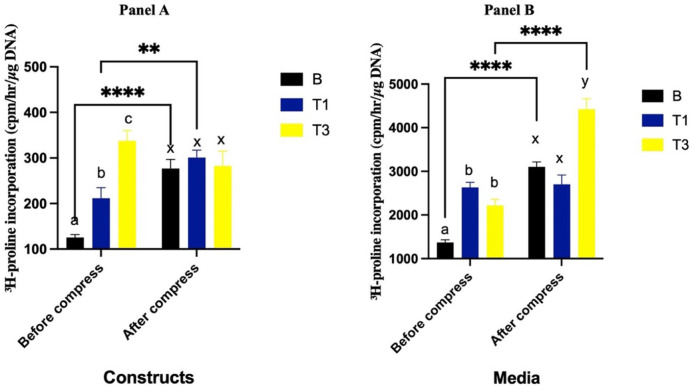
3H proline incorporation (cpm/hr/ μg DNA) into constructs (**Panel A**) and media from constructs (**Panel B**) cultured with Spirulina at 0 (B; carrier only), 30 (T1) and 90 (T3) µg/mL before and after a single bout of dynamic compression. Different lowercase letters (abc: pre-compression, xyz: post-compression) denote significant differences between groups before and after compression. *Indicates significant difference between pre- and post-compression within the same group.

Prior to compression, constructs cultured with T1 and T3 had greater collagen synthesis compared to the B group (*P* = 0.02 and *P* < 0.0001, respectively), with T3 having greater levels than T1 (*P* = 0.0001) (**Fig 4, Panel A**). Following compression, collagen synthesis levels increased in the B (*P* < 0.0001) and T1 constructs (*P* = 0.009). In contrast, T3 constructs did not change after compression (*P* = 0.07). Post-compression, collagen synthesis in T1 and T3 constructs were not different from the B constructs (*P* = 0.62 and *P* = 0.98, respectively).

### Collagen Synthesis in Media

Prior to compression, collagen synthesis in the media was significantly greater in the T1 and T3 groups compared to the B group (*P* < 0.0001 and *P* = 0.003, respectively), with no difference between T1 and T3 (*P* = 0.20). Following compression, collagen synthesis increased in both the B (*P* < 0.0001) and T3 (*P* < 0.0001) groups, whereas no significant change was observed in T1 (*P* = 0.77). After compression, collagen synthesis in T1 was not different than the B group (*P* = 0.18). However, T3 resulted in significantly greater collagen synthesis than both the B (*P* < 0.0001) and T1 (*P* < 0.0001) groups (**Fig 4, Panel B**).

### Collagen Content of Constructs

**Figure 5.**
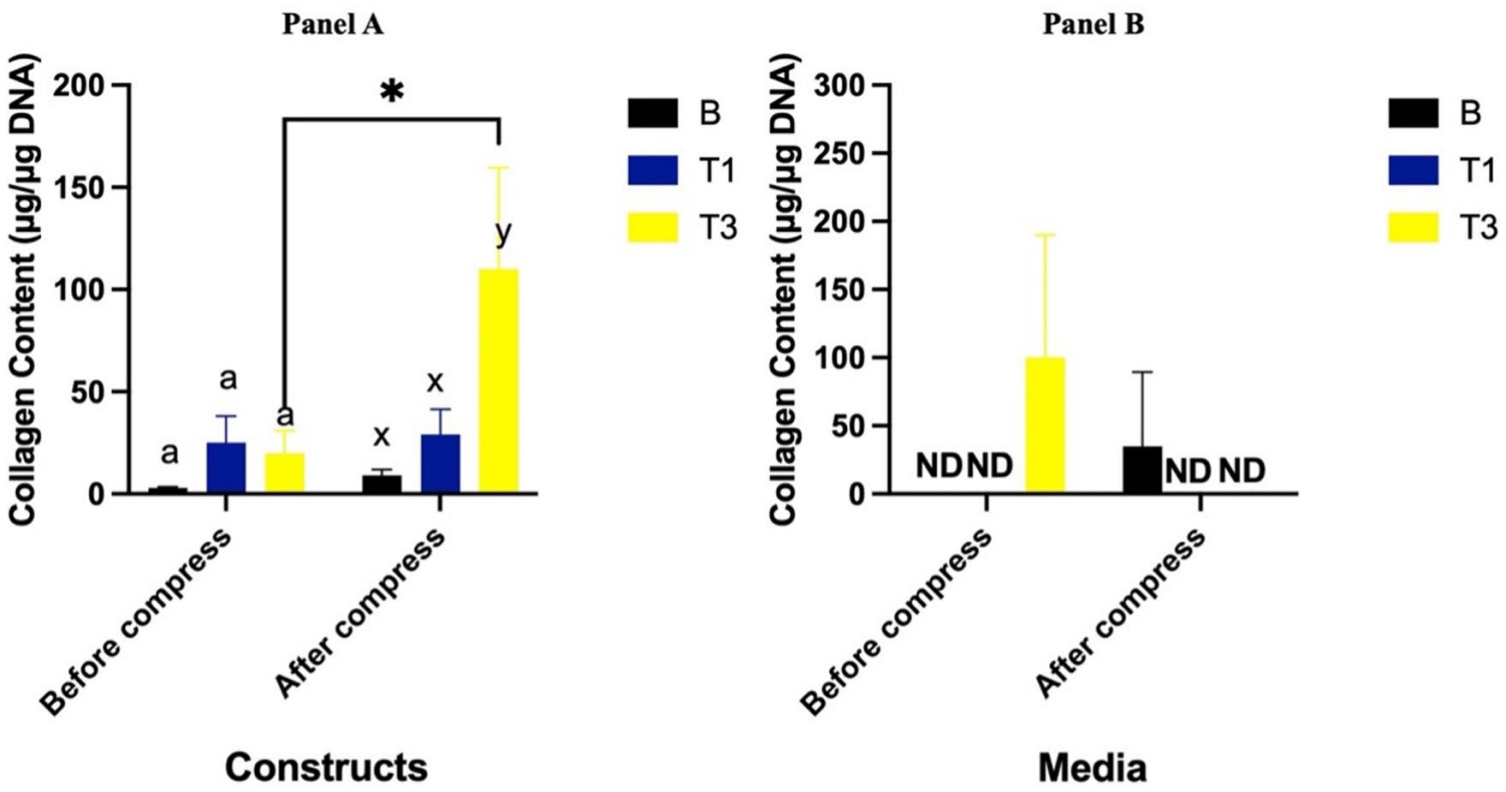
Collagen content (μg/μg DNA) of constructs (**Panel A**) and media from constructs (**Panel B**) cultured with Spirulina at 0 (B; carrier only), 30 (T1) and 90 (T3) µg/mL before and after a single bout of dynamic compression. Different lowercase letters (abc: pre-compression, xyz: post-compression) denote significant differences between groups before and after compression. *Indicates significant difference between pre- and post-compression within the same group. ND indicates not detected.

Prior to compression, collagen accumulation in constructs cultured with T1 and T3 showed no difference compared to the B group (*P* = 0.83 and *P* = 0.90, respectively). Following compression, collagen levels remained unchanged in both the B and T1 groups (*P* = 0.88 and *P* = 0.92, respectively). However, the T3 group exhibited greater collagen content compared to the B group (*P* = 0.03) and showed an increase in collagen concentration from pre- to post-compression (*P* = 0.02) (**Fig 5, Panel A**).

### Collagen Content in Media

Collagen accumulation in the media was not detectable in the B and T1 groups both before and after compression. Prior to compression, collagen levels in the T3 group were detectable but showed no difference compared to B (*P* = 0.15). Following compression, collagen levels in T3 significantly decreased (*P* = 0.02) and became undetectable, similar to the B and T1 groups (**Fig. 5, Panel B**).

## Discussion

This study examined the effects of Spirulina conditioning on chondrocyte-seeded agarose constructs exposed to dynamic compression, focusing on NO production, proteoglycan content and release, and collagen synthesis and retention. The primary findings indicate that moderate dynamic strain (10%) promotes ECM metabolic activity and that Spirulina modulates cartilage homeostasis in a dose-dependent, nonlinear manner. In particular, the high-dose Spirulina group (T3; 90 µg/mL) exhibited the most pronounced responses, including elevated NO production, increased proteoglycan release, enhanced pre-compression collagen synthesis, and greater post-compression collagen content, whereas the low-dose group (T1; 30 µg/mL) less changes. While these findings suggest that Spirulina influences cartilage mechanobiology, the underlying mechanisms and functional implications require careful interpretation within the context of existing literature.

Mechanical loading initiates a cascade of biochemical and biophysical responses in cartilage that influence chondrocyte metabolism and ECM organization. NO is a key mediator within these pathways, contributing to both mechanotransduction and inflammatory signaling.^28,29^ In the present study, dynamic compression increased NO production in untreated constructs, consistent with activation of mechanosensitive signaling pathways under physiological loading.^28,29^ Previous studies have reported that moderate dynamic strain can upregulate NO synthesis, whereas prolonged, static, or injurious loading may suppress NO production or shift signaling toward catabolic pathways.^28,30,31^ Such increases in NO have been reported during adaptive cartilage loading and may facilitate matrix turnover, cytoskeletal reorganization, and load-induced signaling rather than reflecting overt stress.

However, the biological role of NO in cartilage is highly context-dependent. At physiological concentrations, NO participates in mechanoadaptive signaling, regulation of matrix turnover, and maintenance of cellular homeostasis.^32^ Conversely, excessive or sustained NO production, particularly when driven by inflammatory cytokines such as IL-1α or TNF-α, has been strongly associated with oxidative stress, mitochondrial dysfunction, inhibition of matrix synthesis, and upregulation of matrix metalloproteinases (MMPs), contributing to cartilage degeneration and osteoarthritis progression.^33^ Importantly, the present study did not assess NO synthase isoform expression, reactive nitrogen species formation, or oxidative damage markers. As such, the observed increases in NO, particularly in Spirulina-treated constructs, cannot be definitively classified as adaptive or pathological. Instead, these findings should be interpreted as alterations in NO signaling, with functional consequences that remain to be clarified. Accordingly, the role of NO in this system should be considered a working hypothesis requiring further verification rather than a definitive indicator of either protective or deleterious activity.

Proteoglycans are essential for cartilage hydration and compressive function,^34^ and their tissue content reflects a balance between synthesis, degradation, and physical redistribution influenced by mechanical strain and inflammatory signaling.^35,36^ In the present study, proteoglycan content declined following compression in control and low-dose Spirulina constructs, consistent with numerous reports demonstrating that dynamic loading can suppress proteoglycan retention and promote release into the surrounding medium.^37-40^ Importantly, proteoglycan loss under mechanical loading does not necessarily indicate irreversible ECM degradation, particularly in engineered constructs with lower collagen density and higher permeability. Several studies have shown that cyclic compression induces fluid-driven transport of negatively charged GAGs and matrix reorganization, particularly in engineered or immature cartilage constructs with relatively low collagen density.^37,38,40^

From this perspective, the observed proteoglycan release may reflect reversible ECM remodeling driven by interstitial fluid pressurization, altered osmotic balance, and changes in macromolecular crowding.^41-43^ Such physicochemical redistribution has been described as an adaptive response that facilitates matrix reorganization under repeated mechanical deformation.^41-43^ An alternative explanation for the observed GAG release is that moderate dynamic loading and altered NO signaling may have transiently activated aggrecanases (e.g., ADAMTS-4/5) or MMPs, leading to controlled proteoglycan cleavage rather than purely physicochemical redistribution.^44,45^ Transient activation of these enzymes has been reported under moderate dynamic loading and may facilitate matrix reorganization without causing irreversible degradation.^44,45^ Conversely, sustained or excessive activation of the same proteases is a hallmark of pathological cartilage breakdown in osteoarthritis. In the present study, the absence of proportional tissue proteoglycan loss despite increased GAG release—particularly in Spirulina-treated constructs—suggests regulated turnover rather than extensive degradation. However, without direct measurements of enzyme activity, neoepitope formation, or GAG fragment size, this interpretation remains speculative and requires further verification.

Notably, Spirulina-treated constructs (particularly in the T3 group) exhibited increased proteoglycan release without a proportional reduction in tissue proteoglycan content. This pattern suggests that proteoglycan release was not accompanied by extensive net matrix loss, favoring an interpretation of controlled remodeling rather than overt degradation. Nevertheless, this conclusion remains tentative, as physicochemical redistribution and enzymatic turnover are not mutually exclusive processes. Future studies incorporating direct measurements of aggrecanase activity, sulfated GAG fragment analysis, and ECM ultrastructure will be necessary to distinguish between these mechanisms.

Collagen synthesis and retention are key determinants of cartilage structural integrity, and their response to mechanical loading depends on strain magnitude, loading regime, and matrix maturity.^46^ In the present study, collagen content increased following compression in control constructs, consistent with reports demonstrating anabolic responses to dynamic loading in more mature cartilage matrices.^46^ However, interpretation of collagen detected in the culture medium requires particular caution. Thus, increased media collagen should be interpreted as altered collagen metabolism rather than unequivocal evidence of tissue damage.

Spirulina supplementation altered the balance between construct-retained and released collagen in a dose-dependent manner. The low-dose group exhibited greater collagen retention within the constructs, whereas the high-dose group showed elevated collagen levels in the surrounding media alongside increased total collagen synthesis. One interpretation is that higher Spirulina concentrations promote more dynamic collagen turnover, potentially reflecting accelerated remodeling. Alternatively, increased media collagen could also indicate early collagen network disruption, as collagen degradation is a defining feature of progressive cartilage degeneration.^3^ Because collagenase activity, MMP expression, and collagen cleavage products were not assessed, it is not possible to conclusively distinguish between adaptive turnover and pathological breakdown. Accordingly, the presence of collagen in the culture medium should be interpreted as evidence of altered collagen metabolism rather than definitive proof of either beneficial remodeling or degradation.

The dose-dependent effects observed between T1 and T3 indicate a nonlinear biological response to Spirulina supplementation. At the lower dose, Spirulina was associated with modest NO production, limited proteoglycan release, and preferential collagen retention, consistent with a stabilizing ECM profile. At the higher dose, Spirulina induced greater NO signaling, increased matrix redistribution, and enhanced collagen turnover. One potential explanation is that higher Spirulina concentrations increase NO bioavailability by scavenging reactive oxygen species and reducing peroxynitrite-mediated NO inactivation,^47^ thereby prolonging NO-dependent mechanotransductive signaling.^32^ While plausible, this mechanism remains speculative and requires direct experimental validation.

These findings are consistent with our previous work examining simulated Spirulina digestion in cartilage explants exposed to inflammatory challenge, where Spirulina reduced GAG loss while increasing NO production in a dose- and oxygen-dependent manner.^48^ Similar dual effects of NO have been reported in both human and equine exercise studies, where NO contributes to vascular adaptation and recovery but may also participate in oxidative stress under excessive load.^35,49,50^ Collectively, these observations support the concept that Spirulina does not act solely as a passive antioxidant but actively modulates redox-sensitive signaling pathways involved in cartilage mechanobiology.

Although relatively few studies have directly examined Spirulina’s effects on cartilage, accumulating evidence highlights its antioxidant and anti-inflammatory properties in musculoskeletal contexts. Spirulina-derived phycocyanin has been shown to inhibit MMP activity, modulate NO production, and preserve proteoglycan and collagen integrity in osteoarthritis models.^51,52^ Importantly, most prior studies have focused on pathological inflammation, whereas the present work extends these observations to mechanically driven cartilage adaptation.

While this study employed bovine chondrocytes, the mechanobiological pathways examined—NO signaling, proteoglycan turnover, and collagen remodeling—are highly conserved across species. As such, these findings have relevance to human cartilage biology beyond equine or livestock contexts, particularly in the study of load-mediated cartilage adaptation and nutraceutical modulation. Still, applying these findings to whole joints or clinical doses should be done cautiously.

Taken together, these data demonstrates that Spirulina conditioning of cartilage constructs exerts dose-dependent and qualitatively distinct effects on chondrocyte responses to dynamic mechanical loading. While Spirulina clearly alters NO signaling and ECM metabolism, the relative contributions of adaptive remodeling, physicochemical redistribution, and early degradation cannot be definitively resolved within the scope of the current analyses. Consequently, several interpretations advanced here should be regarded as hypotheses requiring further verification. Future studies incorporating direct measurements of oxidative stress, protease activity, mechanotransductive signaling pathways, and ECM ultrastructure will be essential to fully define Spirulina’s role in cartilage mechanobiology and its potential translational applications in tissue engineering and rehabilitation.

## Limitations and Future Directions

This study has several limitations that should be considered when interpreting the findings and that highlight important directions for future research. First, the experimental sample size was limited to 2 independent experiments, which restricts statistical power and increases sensitivity to inter-experimental variability. Replication across additional biological donors and experimental runs will be necessary to confirm the robustness and generalizability of the observed Spirulina- and load-dependent effects.

Second, the cartilage constructs represent a simplified *in vitro* model that does not fully recapitulate the structural, biochemical, and cellular complexity of native cartilage. In particular, the constructs exhibited lower retention of newly synthesized matrix components compared with longer-term cultures and native cartilage (~50–75% vs. 85%–99%),^53^ which may influence interpretation of ECM stability and turnover. Moreover, the absence of interactions with synovial fluid, subchondral bone, vascular factors, and immune cells limits the extent to which inflammatory and adaptive responses observed here can be extrapolated to a real joint environment. In addition, this study utilized bovine chondrocytes, which share many structural and mechanobiological characteristics with human cartilage but differ in metabolic rate, matrix turnover, and responsiveness to nutritional interventions. Consequently, extrapolation of Spirulina dosing and mechanobiological responses to human cartilage should be made with caution and requires validation in human-derived cells or clinically relevant models

Third, mechanistic interpretation is constrained by the limited panel of molecular markers assessed. Catabolic and inflammatory mediators—including MMPs, aggrecanases (ADAMTS), collagenases, cytokines such as IL-6, reactive oxygen species (ROS), and oxidative damage markers—were not measured. As a result, it is not possible to definitively distinguish adaptive ECM remodeling from early degenerative signaling. Future studies incorporating targeted biochemical, molecular, and redox analyses will be essential to clarify the pathways through which Spirulina modulates cartilage mechanobiology.

Fourth, the experimental timeframe captured acute responses to a single bout of mechanical loading and Spirulina exposure. While this design was appropriate for identifying early mechanosensitive changes, longer-term and repeated loading paradigms are required to determine whether Spirulina promotes sustained ECM stabilization, cumulative remodeling, or progressive matrix degradation. In addition, cell viability was assessed indirectly via DNA quantification, which does not distinguish between live, apoptotic, or necrotic cells. Complementary viability and apoptosis assays would provide a more comprehensive assessment of chondrocyte health.

Finally, the mechanical loading we applied was a simple uniaxial sinusoidal compression (10% strain, 1 Hz), while actual joints experience complex, multi-directional, and repetitive forces. As a result, the responses we measured may not reflect the full range of cartilage behavior under physiological conditions. Future investigations should incorporate more physiologically relevant loading regimes, alongside inflammatory co-stimulation (e.g., IL-1β) and hypoxic culture conditions that better mimic the native cartilage microenvironment. Such approaches would enhance the translational relevance of Spirulina supplementation for joint health and tissue engineering applications.

## Conclusion

Spirulina supplementation modulates chondrocyte responses to dynamic mechanical loading in a dose-dependent, nonlinear manner, affecting NO production, proteoglycan retention, and collagen turnover. High doses (T3; 90 µg/mL) promoted dynamic ECM remodeling, with regulated proteoglycan release and increased collagen metabolism, while low doses (T1; 30 µg/mL) favored matrix stabilization. These effects may reflect adaptive mechanotransductive signaling and antioxidant-mediated protection, but interpretations of NO and collagen responses remain hypothetical, as catabolic enzyme activity and oxidative stress markers were not assessed. Overall, Spirulina shows promise as a modulator of cartilage mechanobiology rather than a simple anti-catabolic agent, warranting further investigation in more physiologically relevant models.
